# Polyimide-Based Nanocomposites with Binary CeO_2_/Nanocarbon Fillers: Conjointly Enhanced Thermal and Mechanical Properties

**DOI:** 10.3390/polym12091952

**Published:** 2020-08-28

**Authors:** Alexandra L. Nikolaeva, Iosif V. Gofman, Alexander V. Yakimansky, Elena M. Ivan’kova, Ivan V. Abalov, Alexander E. Baranchikov, Vladimir K. Ivanov

**Affiliations:** 1 Institute of Macromolecular Compounds, Russian Academy of Sciences, 199004 Saint Petersburg, Russia; gofman@imc.macro.ru (I.V.G.); yakimansky@yahoo.com (A.V.Y.); ivelen@mail.ru (E.M.I.); i.abalf@yandex.ru (I.V.A.); 2 Institute of Chemistry, Saint Petersburg State University, 198504 Saint Petersburg, Russia; 3 Kurnakov Institute of General and Inorganic Chemistry of the Russian Academy of Sciences, 119991 Moscow, Russia; a.baranchikov@yandex.ru (A.E.B.); van@igic.ras.ru (V.K.I.)

**Keywords:** nanocomposites, polyimides, binary nanofillers, nanoceria, carbon nanofibres, carbon nanocones/discs

## Abstract

To design novel polymer materials with optimal properties relevant to industrial usage, it would seem logical to modify polymers with reportedly good functionality, such as polyimides (PIs). We have created a set of PI-based nanocomposites containing binary blends of CeO_2_ with carbon nanoparticles (nanocones/discs or nanofibres), to improve a number of functional characteristics of the PIs. The prime novelty of this study is in a search for a synergistic effect amidst the nanofiller moieties regarding the thermal and the mechanical properties of PIs. In this paper, we report on the structure, thermal, and mechanical characteristics of the PI-based nanocomposites with binary fillers. We have found that, with a certain composition, the functional performance of a material can be substantially improved. For example, a PI containing SO_2_-groups in its macrochains not only had its thermal stability enhanced (by ~20 °C, 10% weight loss up to 533 °C) but also had its stiffness increased by more than 10% (Young’s modulus as high as 2.9–3.0 GPa) in comparison with the matrix PI. In the case of a PI with no sulfonic groups, binary fillers increased stiffness of the polymer above its glass transition temperature, thereby widening its working temperature range. The mechanisms of these phenomena are discussed. Thus, this study could contribute to the design of new composite materials with controllable and improved functionality.

## 1. Introduction

Aromatic polyimides (PIs) exhibiting extremely high thermal and chemical stability, as well as outstanding mechanical and insulating properties, have found widespread use in numerous applications spanning from microelectronics to aerospace engineering [1,2,3]. However, the scope of PIs’ application could be substantially expanded, taking into account the cutting-edge techniques for fabrication of polymer-based nanocomposites doped with various inorganic species, i.e., metal oxides, Fe_3_O_4_ [4], SnO_2_ [5], NiO [6], TiO_2_ [7], Al_2_O_3_ [8], etc. An important advantage of nanocomposites in comparison with conventional multicomponent materials is that nanoparticles provide an increase in the matrix-filler contact area. This opens up the possibility of controlling the interaction parameters through the proper selection of the chemical identity of both a filler and a matrix, the size of the nanoparticles, their concentration and non-stoichiometry, etc. Moreover, the synergistic effect of a PI and nanoparticles can endow a nanocomposite material with considerably improved functional properties.

One of the most extensively studied types of metal oxide nanoparticles is cerium oxide CeO_2_ [9,10,11]. Due to the high mobility of surface oxygen species in nanoscale CeO_2_, cerium can easily alter its oxidation state from Ce(IV) to Ce(III). Hence, ceria nanoparticles show remarkable catalytic activity. The ability of CeO_2_ to release and take up oxygen facilitates its application in the processes where oxygen plays a decisive role, e.g., in vehicle exhaust emission control [12], in solid-oxide fuel cells [13] and even in the protection of living cells against the detrimental effects of UV-radiation [14] and oxidative stress [15]. Furthermore, nanoceria affects a number of physico-chemical properties of polymers of technical use, viz thermal stability [16], mechanical strength [17], optical absorbance [18], etc. 

In our previous work [19,20] we have investigated the impact of CeO_2_ nanoparticles on the thermal properties of a number of polyimides. The thermal stability of a nanocomposite was found to be determined by the structure of a matrix PI, viz only the PIs with SO_2_-groups in the repeating subunits of macromolecules demonstrated a considerable enhancement in thermo-oxidative stability upon doping with ceria nanoparticles. Conversely, the thermo-oxidative stability of the PIs with no sulfonic groups deteriorated upon incorporation of CeO_2_ nanoparticles into the polymer matrix. The effect of nanoceria concentration on the thermal properties of PIs has also been demonstrated [19]. We have proposed a conceptual interpretation of the data obtained based on the specific interactions of the PIs and CeO_2_ nanoparticles [20]. 

Several research studies have addressed the impact of ceria nanoparticles on the mechanical properties of various polymers [17,21]. However, the literature on this issue with regard to PI matrices is still very scarce and controversial [19,22]. Moreover, no information has been found on the simultaneous enhancement of both thermal and mechanical properties. 

The outstanding mechanical characteristics of carbon nanospecies, such as high tensile strength and Young’s modulus, make them one of the most promising reinforcements for polymer-based nanocomposites [23,24], including those based on PI matrices. A plethora of carbon nanoparticles have already been embedded into the PI matrices, e.g., carbon nanotubes (CNT) and nanofibres (CNF) [25], carbon nanocones/discs (CNC) [26], etc., as well as pretreated (chemically modified) carbon nanoparticles [27]. Moreover, carbon nanospecies have been shown to enhance the thermal stability of PIs [26]. However, this positive effect is not that pronounced, as in the case of PI-based nanocomposites containing CeO_2_ nanoparticles [19,20]. 

It is also worth mentioning that the impact of any type of nanoparticles on the physical, in particular thermal and mechanical, properties of polymers depends strongly on a number of factors, viz the size of nanoparticles and their aspect ratio, as well as dispersion degree and concentration, etc. 

The idea of the combination of various types of nanofillers seems quite reasonable, as each of the nanocomponents can improve a certain group of properties [28,29,30]. Obviously, one could expect a simultaneous improvement in both the thermal and mechanical properties of PI-based nanocomposites doped with a binary CeO_2_/nanocarbon filler, due to the synergistic effect of the individual nanoadditives.

In the present paper, we report on an investigation into the impact of binary CeO_2_/nanocarbon fillers on the thermal and mechanical properties of a number of PIs. Two types of nanocarbon have been introduced into the polymer matrices along with the cerium oxide nanoparticles: CNF and a CNC blend. The choice of these two nanofillers was determined by the following prerequisites. The former component had long been used as a reinforcing agent in polymer-based composite materials [31,32], including PI-based ones [25,33], because of its high strength and elastic modulus, whilst the latter nanofiller, also possessing good mechanical and thermal properties, had been reported [26] to impart greater stiffness to nanocomposites at lower (in comparison to CNF) concentrations. Moreover, CNC are barely prone to aggregation up to a certain content of the nanofiller in a matrix, allowing for the introduction of higher amounts of reinforcing nanoparticles [26].

We obtained data on the thermal stability indices of nanocomposites, along with their elastic, strength and deformation characteristics. We also studied the thermo-mechanical behaviour of the nanocomposites, e.g., we tackled the issue of how the addition of nanoparticles (both individual and binary) into the PIs matrices affects their glass transition temperatures (*T*_g_), as well as their softening processes above the *T*_g_. To the best of our knowledge, the approach of a concerted modification of mechanical and thermal properties of composite films based on PIs, has not been employed so far; thereby, our study paves a new way for the improvement of the functional characteristics of polymer-based nanocomposites. The information given in this paper can undoubtedly be useful insofar as it provides a protocol for the fabrication of a series of new polymer-inorganic materials with improved functional characteristics, capable of stable and efficient work under certain operating conditions.

## 2. Materials and Methods 

### 2.1. Materials

**Figure 1:**
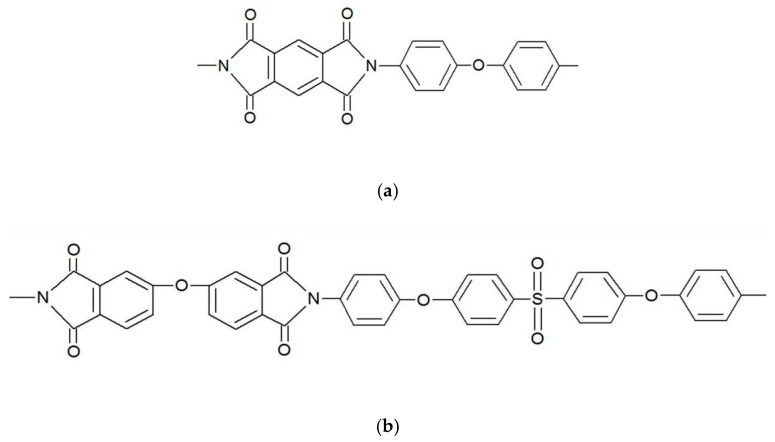
Structural formulas of elementary units of the polyimides (PIs) used in the present investigation. (**a**) PMDA-ODA (PI with a repeating unit based on pyromellitic dianhydride (PMDA) and oxydianiline (ODA)); (**b**) DPhO-BAPS (PI with a repeating unit based on 2,3,3′,4′-diphenyl ether tetracarboxylic acid dianhydride (dianhydride DPhO) and 4,4′-bis(4″-aminophenoxy)biphenyl sulfone (diamine BAPS)).

Thermally stable aromatic PIs (Figure 1) have been used both as polymer matrices for nanocomposites and as reference pristine materials. Based on the experimental data obtained in our previous works [19,20], we chose two PI matrices: one of them contained SO_2_-groups and the other had no sulfonic groups. A two-stage solution technique [26,34] was applied to process both of the PI-based films from the corresponding solution of poly(amic acids) (PAAs) in N-methyl-2-pyrrolidone. The PAA for DPhO-BAPS (PI with a repeating unit based on 2,3,3′,4′-diphenyl ether tetracarboxylic acid dianhydride (dianhydride DPhO) and 4,4′-bis(4″-aminophenoxy)biphenyl sulfone (diamine BAPS)) was synthesised at the Institute of Macromolecular Compounds (RAS) and the PAA for PMDA-ODA (PI with a repeating unit based on pyromellitic dianhydride (PMDA) and oxydianiline (ODA)) was purchased from Sigma Aldrich (St. Louis, MI, USA).

Isotropic CeO_2_ nanoparticles (5–6 nm) were prepared by a solution processing method described elsewhere [35] involving mixing cerium(III) nitrate (0.08 mol/L) dissolved in a water/isopropanol (1:1) solvent with aqueous ammonia (3 mol/L, 900 mL), followed by vigorous stirring of the mixture for 3 h. The yellow precipitate was then washed with distilled water and dried at 60 °C.

Carbon nanofibres (so-called vapour-grown carbon fibres) were purchased from Sigma Aldrich (St. Louis, MI, USA) and annealed at 150 °C (for 1 h) to remove adsorbed moisture. Carbon nanocones/discs were produced by the n-TEC company (Norway). This material consisted of ~20 wt% of carbon cones, ~70 wt% of carbon discs and ~10 wt% of carbon black (impurities). 

### 2.2. Films Preparation

To prepare PI-based nanocomposites (either two- or three-component) we applied the following technique [26]. Dispersions of nanoparticles (nanoceria, nanocarbon or their mixture) in N-methyl-2-pyrrolidone, preliminarily sonicated for 1 h, were mixed with corresponding PAA solutions and stirred for 24 h so as to provide a quasi-homogeneity of the systems. Then, the resulting compositions were cast on glass supports and dried for 4 h at 80 °C, the same being done with the reference pristine PAA solutions. The final curing of the films obtained was carried out by gradual heating up to 365 °C for PMDA-ODA and 300 °C for DPhO-BAPS, followed by treatment at these temperatures for 30 min. 

### 2.3. Characterization Techniques

To investigate and to compare the structures of the fabricated samples, scanning electron microscopy (SEM) images of their cryo-cleavages were recorded using a SUPRA-55VP scanning electron microscope (Carl Zeiss, Oberkochen, Germany) equipped with a secondary electron detector, as well as a detector of back-scattered electrons. The samples were fixed on the microscope holders with a special glue and coated with a thin layer of platinum. Additionally, X-ray diffraction (XRD ) analysis of films of the matrix polymers and their nanocomposites was carried out using a D2 Phaser Bruker AXS (CuKα) X-ray diffractometer. The registration of X-ray beam diffraction was performed in reflection mode.

A DTG-60 setup (Shimadzu, Kyoto, Japan) capable of simultaneous thermogravimetric (TGA) and differential thermal analysis (DTA) was used to study the thermal properties of the materials, the samples being heated to 600 °C at a rate of 5 °C/min in air flow (80 mL/min). Using TGA curves, we determined the thermal stability indices of the samples, *τ*_5_ and *τ*_10_ (the temperatures at which a polymer or a composite loses 5% and 10% of its initial weight, respectively, due to thermal destruction processes). DTA curves were used to determine the *T*_g_ values of the samples and identify their supramolecular structure.

The glass transition temperatures of the PI-based compositions were also measured by the thermomechanical method (TMA), using a TMA 402 F1 Hyperion thermal analyser (NETZSCH, Selb, Germany). The rate of sample heating was 5 °C/min. All experiments were carried out in argon flow (70 mL/min).

Mechanical tests of the films were performed in the uniaxial extension mode using an AG-100kNX Plus universal mechanical test system (Shimadzu, Kyoto, Japan). Experiments were conducted at room temperature. Strip-like samples 2 × 30 mm in size were stretched at a rate of 10 mm/min, according to ASTM D638 requirements. The Young’s modulus *E*, the yield stress *σ*_y_, the break stress *σ*_b_, and the ultimate deformation *ε*_b_ were determined.

## 3. Results and Discussion

### 3.1. Optimization of Composition

**Figure 2:**
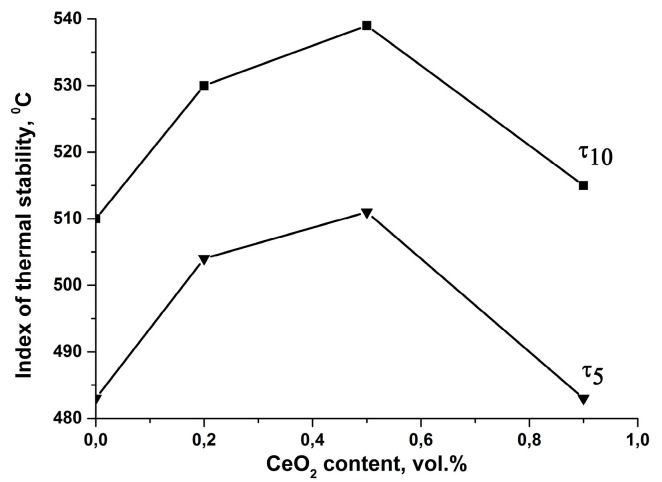
Thermal stability indices *τ*_5_ and *τ*_10_ of the DPhO-BAPS-CeO_2_ nanocomposites with various content of the nanofiller.

In order to determine an optimal nanoceria concentration for the enhancement of DPhO-BAPS thermal stability, we preliminarily carried out a number of TGA runs on DPhO-BAPS/CeO_2_ nanocomposites with various contents of the nano-additive. The results are shown in Figure 2. It is obvious that 0.5 vol% of the nanoceria imparted the highest thermal stability to the DPhO-BAPS. Henceforth, we used this optimal concentration in all the subsequent experiments. Although we had shown previously that nanoceria reduced the thermal stability of PMDA-ODA at any concentration of the nanoparticles up to 0.9 vol% [19], we chose the same CeO_2_ concentration (0.5 vol%) for the PMDA-ODA/CeO_2_ compositions so as to facilitate comparison of the thermal, mechanical, and thermo-mechanical properties of all the samples under study.

### 3.2. Investigation of Structure and Morphology

**Figure 3:**
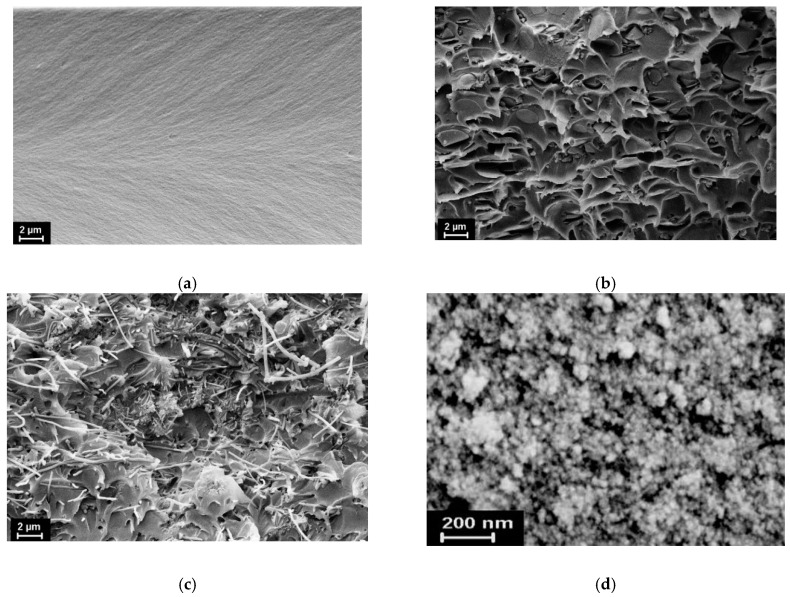
Scanning electron microscopy (SEM) images of (**a**) pristine DPhO-BAPS matrix; (**b**) nanocomposite based on DPhO-BAPS doped with CeO_2_/CNC (carbon nanocones) (3 vol%); (**c**) nanocomposite based on DPhO-BAPS doped with CeO_2_/CNF (carbon nanofibres)(3 vol%); (**d**) pristine CeO_2_ powder.

To confirm the successful distribution of nanoparticles within the polymer matrices, SEM images of the samples’ cryo-cleavages were obtained (Figure 3). 

A SEM image of the pristine CeO_2_ powder is also shown in Figure 3. It was found that doping the polymer matrices with carbon nanoparticles drastically changed the morphology of their fracture surfaces. Both the CNC and the CNF were homogeneously dispersed in the PI matrix, in spite of some aggregation of the latter nanoparticles. Using a backscattered electrons detector (sensitive to the atomic number of chemical elements), we registered almost no CeO_2_ agglomerates with a size of tens of nanometres or bigger. This indirectly confirmed the uniformity of nanoceria distribution within the polymer matrix, at least on the scale considered. Small aggregates of sphere-like nanoparticles observed in Figure 3b are assumed to be amorphous carbon (from the pristine CNC powder), since they did not manifest a noticeable difference in the intensity of backscattering with regard to the surrounding species. In spite of the fact that we could not detect a mono-dispersed size distribution of nanoceria in the samples, we did register certain pronounced effects, including synergistic effects, on the functional properties of the polymer matrices under study. 

**Figure 4:**
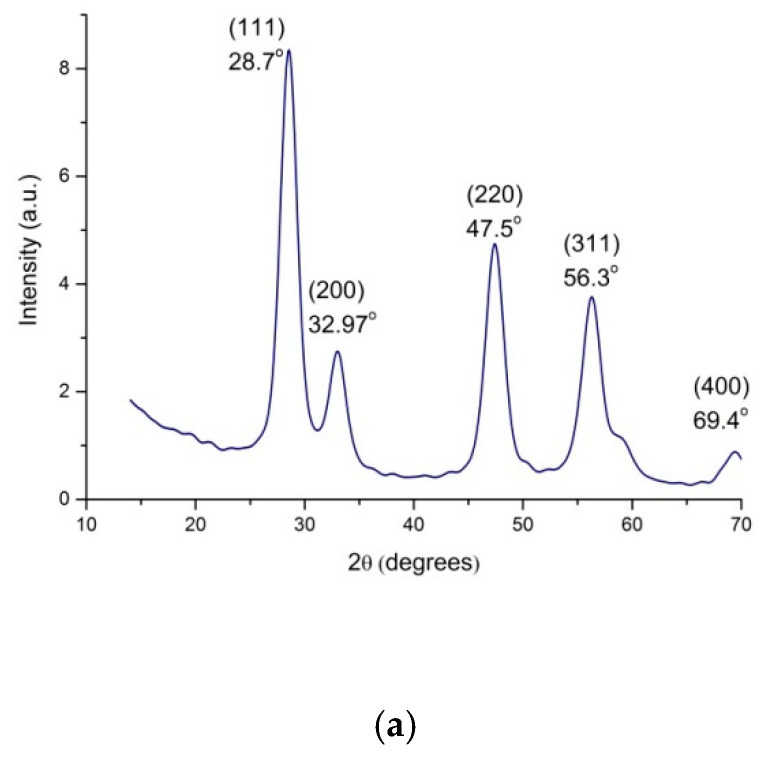

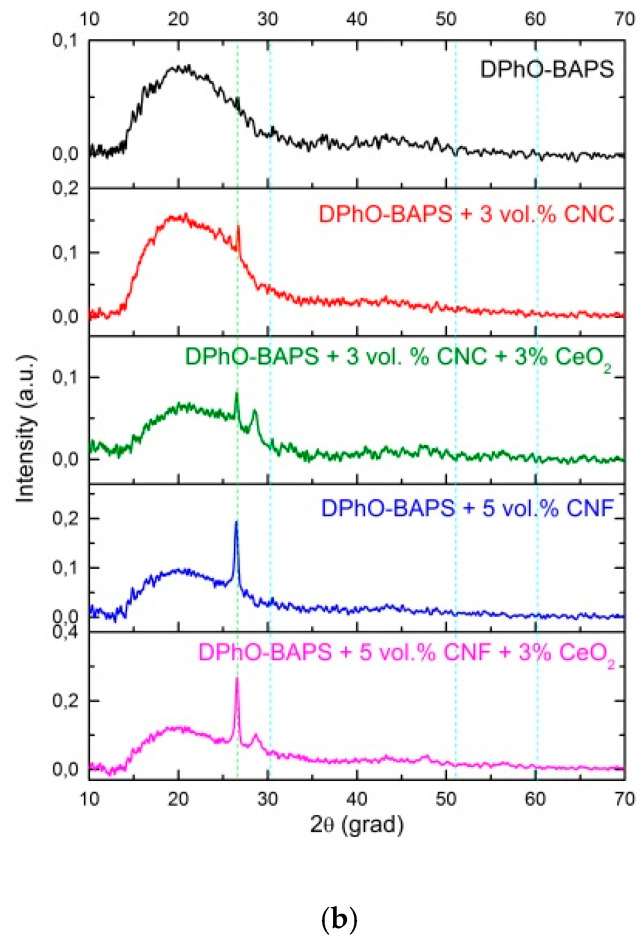
X-ray diffraction (XRD) patterns of (**a**) pristine CeO_2_ powder and (**b**) DPhO-BAPS matrix, as well as its nanocomposites with various combinations of nanoparticles.

Figure 4 shows XRD patterns of the pristine CeO_2_ powder and the DPhO-BAPS matrix, as well as of the DPhO-BAPS–based nanocomposites with various fillers.

The diffraction pattern of a pristine CeO_2_ powder exhibits sharp reflections at 2θ = 28.7°, 32.97°, 47.5°, and 56.3°, which are attributed to (111), (200), (220), and (311) planes, respectively, and correspond to the cubic fluorite crystal structure (ICDD PDF card #34-394, data from NIST (National Institute of Standards and Technology, USA)). The full width at half-maximum (FWHM) of the (111) peak was used for calculation of the apparent transverse D_111_ sizes of the CeO_2_ crystals by Scherrer’s equation [36]. A D_111_ size was found to be ~5.7 nm. The XRD pattern of PI shows a broad diffraction peak resulting from diffuse scattering on the amorphous polymer. This single peak remains in the diffraction patterns of all the nanocomposites. It implies that incorporation of nanoparticles into the PI did not change the amorphous structure of DPhO-BAPS.

Diffraction patterns of all nanocomposite samples contain a diffraction maximum at 2θ = 26.5° corresponding to diffraction from (002) planes of graphite. The DPhO-BAPS systems with binary CeO_2_/nanocarbon fillers display a set of low-intensity reflections peaking at 2θ = 28.7°, 47.5°, and 56.3°, which are attributed to the CeO_2_ cubic structure.

### 3.3. Thermal Properties

**Figure 5:**
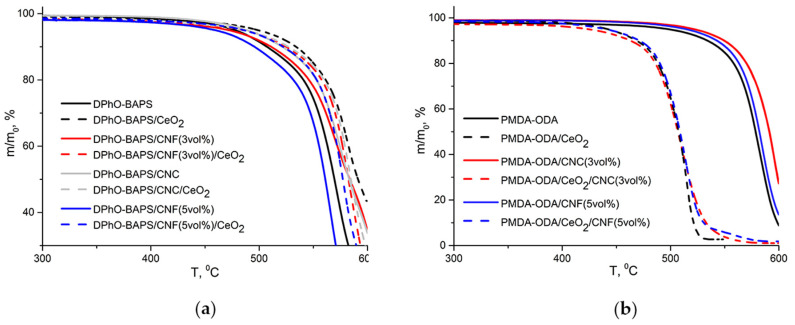
Analysis (TGA) curves of (**a**) DPhO-BAPS-based samples; (**b**) PMDA-ODA-based samples.

The thermal properties of the nanocomposites, as well as of the reference samples, were investigated with simultaneous TGA and DTA. The TGA curves of both of the PIs and the corresponding nanocomposites are plotted in Figure 5; their thermal stability indices are summarised in Table 1.

Almost all low-molecular weight compounds (water and solvent residues) were removed during the films processing, since only 2 wt.% loss was observed up to a temperature of 300 °C (see Figure 5). It is clearly seen that all of the samples under study showed one-stage thermal degradation, the CeO_2_ impact on the thermal behaviour of the two PI matrices being completely different. A PI containing SO_2_-groups had its thermal stability improved after being doped with nanoceria (*τ*_10_ of DPhO-BAPS increased by ~30 °C; see Table 1), while a PI with no sulfonic groups lost its thermal stability (*τ*_10_ of PMDA-ODA decreased by 70 °C). 

These observations are in agreement with the data we have reported earlier [19,20]. In [20], we proposed a possible mechanism for the CeO_2_-PI interactions bringing about the improvement/deterioration of PIs’ thermal stabilities. Briefly, it was supposed that active oxygen species (oxide, peroxide, superoxide, etc.) formed on the surface of nanoparticular CeO_2_ during PI heating in an air atmosphere could participate in a number of chemical reactions, including deprotonation of a benzene ring, followed by homolytic scission of chemical bonds in polymer molecules, with subsequent oxidation of the destruction products and evaporation of volatile substances. However, considering the molecular structure of SO_2_-containing PIs, we presumed that a sulfonic radical (supposedly formed at the initial step of the PIs’ thermal decomposition) with a positive charge on a sulfur atom was electrostatically attracted to the CeO_2_ surface with a negatively charged oxygen species adsorbed on it, thereby decreasing a number of volatile products in a definite temperature range. In this case, the thermal stability of the PI was improved.

It is also evident from Figure 5 that not only nanoceria, but also the carbon nanoparticles, imparted better thermal stability to the PI matrices. This corresponds exactly to literature data [26,37]. In the case of CNF, such an effect could be attributed to the formation of a network-like structure by the nanofiller. This network reduces the out-diffusion speed of the PI degradation products. Considering a graphene-like honeycomb structure of the CNC [38], one could anticipate specific interactions between their hexagonal network and aromatic rings, as well as heterocycles of the PI macromolecules. This endows the polymer matrix with additional crosslinks, which in turn enhance its thermal stability.

The introduction of the binary CeO_2_/nanocarbon fillers into the DPhO-BAPS matrix led to an increase in its thermal stability (Figure 5a), the latter being in between those of the pristine PI and the nanocomposite filled with the nanoceria only. Apparently, despite the fact that the carbon nanoparticles could act as additional links between the polymer molecules, they also hindered the contact of the macromolecular radicals (being formed during the thermal degradation process) having terminal SO_2_-groups with the CeO_2_ nanoparticles. This resulted in a slight decrease in the thermal stability indices of the DPhO-BAPS doped with the binary nanofillers in comparison with those of the CeO_2_-doped sample.

Using DTA, we determined *T*_g_ values for the DPhO-BAPS-based samples (for ease of comparison with data obtained from TMA we provided the *T*_g_ values in Section 3.4.2). The *T*_g_ of the pristine polymer was slightly higher than those of all the corresponding nanocomposites. One could speculate that a nano-additive hampers interactions between the polymer chains, thereby causing an increase in its segmental mobility.

### 3.4. Thermomechanical Properties

#### 3.4.1. PMDA-Based Compositions

The *T*_g_ values for the PMDA-ODA-based samples containing nanoceria could not be accurately retrieved from the DTA curves, since the temperature intervals of glass transition and decomposition of the polymer partially overlapped. Hence, we employed TMA to determine the said *T*_g_ values. The DPhO-BAPS-based samples were also studied by TMA for comparison purposes.

It should be pointed out that all the samples under investigation had an amorphous structure, since no endothermic peaks indicating the melting of the crystalline phase were registered in the DTA curves.

**Figure 6:**
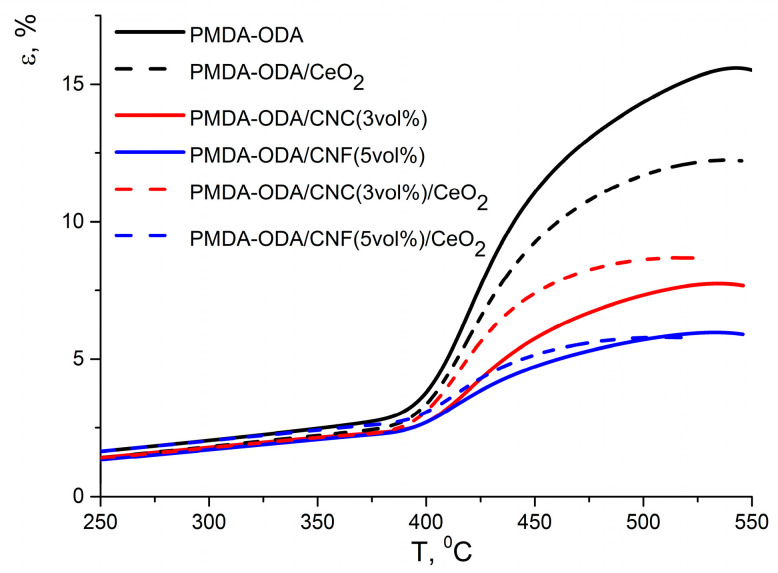
Thermomechanical analysis (TMA) curves of the PMDA-ODA and the corresponding nanocomposites.

**Figure 7:**
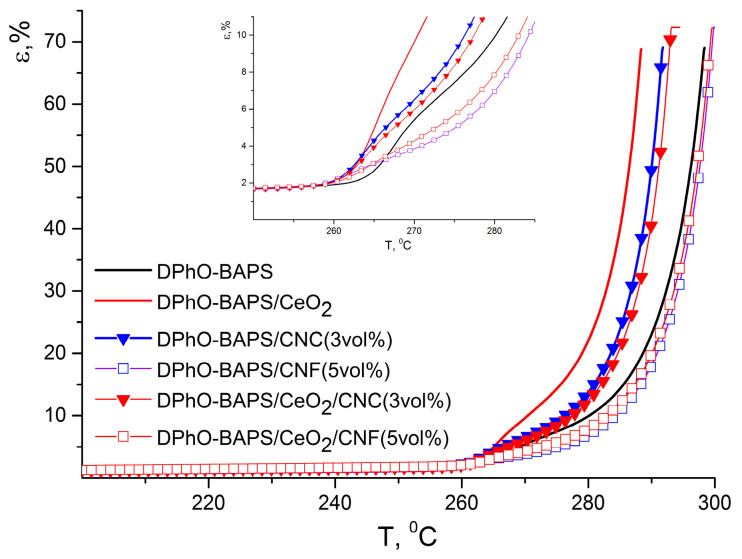
TMA curves of DPhO-BAPS and the corresponding nanocomposites (inset shows the thermal behavior of the samples at small deformations).

Two different stress modes, viz 0.5 MPa and 25 kPa for PMDA-ODA and DPhO-BAPS, respectively, were applied in TMA, since the two PI matrix polymers differ significantly in thermomechanical behaviour (see Figure 6 and Figure 7). We used a standard stress of 0.5 MPa with respect to the pristine and the nanocomposite films of PMDA-ODA, given that this polymer is semi-flexible, although some ordering of its macromolecules can occur during film processing [34]. Since PMDA-ODA has quite a high *T*_g_ (usually higher than 350 °C), it starts to decompose rather quickly above the glass transition temperature. According to Reference [34,39], the decomposition process is accompanied by the formation of so-called «destruction crosslinks» between the PI macromolecules. This results in retardation of the PI chains’ segmental mobility at temperatures higher than *T*_g_, while the PMDA-ODA films reveal quite modest elongation, even above the *T*_g_. The destruction processes in materials are known to be more pronounced in an oxidising air atmosphere; hence, we carried out the TMAs in argon flow so as to make the glass transition of all of the samples in Figure 6 and Figure 7 more evident. 

It can be seen in Figure 6 that, above the *T*_g_, the pristine PMDA-ODA film exhibited a considerable elongation, however, the deformation rate decreased gradually when the temperature reached 430 °C, implying an initial stage of PI thermal degradation followed by the formation of destruction crosslinks [34,40]. The latter are regarded to decrease the mobility of PMDA-ODA macromolecules. The more crosslinks that are formed up to a certain temperature, the slower a material’s compliance grows on heating. A value of the deformation increase, Δ*ε* (see Table 2), in the temperature range from *T*_g_ to a temperature corresponding to a maximum of the TMA curve, may serve as a characteristic of the crosslinking.

The impact of doping PMDA-ODA with various fillers on its thermomechanical behaviour can be estimated using Table 2. One can observe that the *T*_g_ of this PI changed only slightly, to lower values (except for a composite with the CNC filler). The decrease could have stemmed from a hindrance to interactions between the PI macromolecules caused by nanoparticles. On the other hand, the shape of the PMDA-ODA TMA curve above *T*_g_ was strongly affected by the presence of nanoparticles (see Figure 6). All of the nanoparticles introduced into the polymer matrix are likely to have an effect on the segmental mobility of the PMDA-ODA macromolecular chains, decreasing its flexibility. It can be seen from Table 2 that Δε of the unfilled PMDA-ODA film exceeded 12%, which corresponds to a fairly modest stiffness. This hinders the applicability of the PI film above *T*_g_. For all the PMDA-ODA-based nanocomposites, Δε values were noticeably lower than those of the matrix PI. The smallest alteration in Δε as compared to the pristine PI films (9.6%) was registered for the composite filled with CeO_2_ nanoparticles. The most pronounced change came about in the case of CNF-containing nanocomposites, their stiffness below *T*_g_ and above *T*_g_ being almost the same, (Δε for these materials did not exceed 3.6%). It should be noted that, according to Reference [20,39], neither CeO_2_ nanoparticles nor nanocarbon affect the thermal stability of PMDA-ODA in an inert atmosphere; therefore, the increase in stiffness of all the nanocomposites is supposedly not related to the acceleration of the formation of the destruction crosslinks within the PI. However, nanoparticles may well take part in crosslinking and, being quite large, impede the motion of macromolecular segments. Thus, CeO_2_ nanoparticles, having sizes much smaller than those of CNC, influence PI stiffness less than CNC. Apparently, a network of entangled CNF decreases segmental mobility (thereby augmenting the stiffness of a nanocomposite) in an even more salient way.

From a practical point of view, this means that such nanocomposites could be used, even at temperatures higher than *T*_g_ (actually up to *T* of degradation), without noticeable changes in their mechanical properties, provided that the applied stress is not very high.

#### 3.4.2. DPhO-BAPS-Based Compositions

As mentioned above, the thermomechanical behaviour of the DPhO-BAPS-based samples differs fundamentally from that of the PMDA-ODA and its nanocomposites. Preliminary tests for the DPhO-BAPS-based films showed that the matrix polymer, as well as the corresponding nanocomposites, demonstrated a sharp transition to a plastic state. Even under very little stress, the films stretched far beyond the range of deformations registered by the setup. Hence, we applied a 25 kPa stress instead of the standard 0.5 MPa. The TMA curve of the pristine DPhO-BAPS, unlike that of the PMDA-ODA, has three regions (see Figure 7). 

The first one corresponds to a glassy state, with small deformations at moderate stresses taking place. It should be mentioned that DPhO-BAPS, possessing a number of bridge groups between aromatic rings in the repeating unit, which impart high flexibility to the polymer molecules, had a *T*_g_ that was much lower than that of the PMDA-ODA (see Table 3). The temperature region above the *T*_g_ is related to an elastic state of the polymer. The higher the temperature, the more plastic deformations superpose on elastic deformations. At temperatures ~20–25 °C above *T*_g_, the relative motion of macromolecular chains as a whole becomes so eased that the polymer starts «flowing» (corresponding temperatures are denoted by *T*_fl_). Such plastic behaviour of the DPhO-BAPS is explained by its supra-molecular structure, rather than by the structure of its individual macromolecules. The point is that, unlike pre-polymers of many other aromatic PIs, that of the DPhO-BAPS maintains its genuinely amorphous structure with disorderly packed macromolecular coils (inherent to a liquid solution) upon transition to a condensed state [41]. Such a structure partially remains in the resulting PI after the curing process. This facilitates the transition to a plastic state as temperature increases.

Doping DPhO-BAPS with all of the nanoparticles reduced its *T*_g_ only slightly (see Table 3). The same situation was observed for PMDA-ODA nanocomposites (see Table 2). Both the *T*_g_ of the pristine DPhO-BAPS and the changes in this characteristic caused by the introduction of the nanoparticles are in good agreement with DTA data (see Table 3).

Considering the effect of nanoparticles on the thermomechanical behaviour of DPhO-BAPS, it can be seen that CeO_2_ and CNC hardly affected the material’s compliance in the temperature range from *T*_g_ up to *T*_fl_ (Figure 7). This compliance can be characterised by the d*ε*/d*T* value. For the matrix polymer, as well as for the composites containing nanoceria, CNC or their combination d*ε*/d*T* in this temperature interval equal 0.75 ± 0.15%/deg.

A more palpable effect on the thermal behaviour of DPhO-BAPS above *T*_g_ was detected upon addition of CNF into this PI. The flexibility of the composites filled with the CNF, as well as with a binary CNF/CeO_2_ mixture, increased considerably slower than that of the other nanocomposites studied (d*ε*/d*T* = 0.21 ± 0.04%/deg). This indicated a slower rate of increase in segmental mobility of the former nanocomposites beyond *T*_g_. This is probably due to restriction of the PI macrochains’ motion caused by the presence of the nanofibres. The said discrepancy between the behaviour of the CNF-containing nanocomposites and that of the other nanocomposites implies a certain difference in the processes of destruction of intermolecular bonds which took place in the course of glass transition [42]. 

The addition of nanoceria into the DPhO-BAPS entailed a slight decrease in the temperature of transition to a plastic state for the material (see *T*_fl_ in Table 3). It probably resulted from a partial blocking of the PI macrochains’ interaction by the ceria nanoparticles. This effect was presumably not compensated by the formation of any new polymer-nanoparticle bonds in this composite. In turn, the CNF widened the temperature range of the elastic state of the nanocomposite, since the nanofibres were supposed to form a firm network which blocks the motion of the PI macromolecules. This not only shifted the *T*_fl_ to higher values but also decreased the relative deformation of the material in the elastic state (e.g., at *T* = 275 °C, ε of the pristine DPhO-BAPS was 8%, while that of the DPhO-BAPS/CNF/CeO_2_ amounted to only 5%; see Figure 7). From a practical standpoint, it means that such a nanocomposite could maintain its stiffness and operate at temperatures even higher than *T*_g_, at which the pristine PI would start flowing. It should be pointed out that the flexibility of all the DPhO-BAPS-based films increased at the same rate as temperature rose above *T*_fl_ (Figure 7).

### 3.5. Mechanical Properties

**Figure 8:**
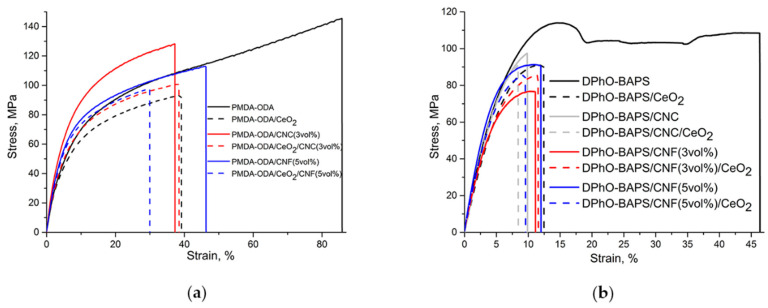
Stress-strain curves of the samples: (**a**) PMDA-ODA-based compositions; (**b**) DPhO-BAPS-based compositions.

As mentioned above, the widespread and increasing use of polymer materials, including PI ones, is determined substantially by their excellent mechanical properties, i.e., by a combination of high strength with the capability of reversible deformations. Table 4 and Table 5 show the mechanical properties of the PMDA-ODA- and DPhO-BAPS-based samples, respectively. The stress-strain curves of all the materials studied are given in Figure 8.

It is evident that, in the stress-activated elastic state (above the yield point), the pristine PIs behaved differently. The PMDA-ODA film exhibited uniform elongation, with no visible localisation of deformation (see Figure 8a). In contrast, the deformation process in the DPhO-BAPS film was strongly non-uniform. There is a well-defined yield point as a local maximum on the DPhO-BAPS stress-strain curves in the strain range of about 15% (Figure 8b), with a so-called «neck» being formed on the PI film. In general, subsequent deformation of a film takes place due to the propagation of a neck throughout the sample. However, some oscillations on the DPhO-BAPS curve can be seen in Figure 8b, and these are attributable to the formation of additional necks merging into one during the process of film elongation.

Figure 8 also shows the mechanical properties of PI samples filled with different types of nanoparticles. Obviously, in both PIs under study, the introduction of CeO_2_ nanoparticles did not lead to any enhancement of the mechanical characteristics of the materials (see also Table 4; Table 5). Their stiffness and strength deteriorated (*E* value decreased by 8 and 20% for DPhO-BAPS- and PMDA-ODA-based nanocomposites, respectively, while *σ*_b_ value decreased by 35 and 14% for these materials). The yield point for the DPhO-BAPS/CeO_2_ nanocomposite could not even be reached. This fact is not surprising, taking into account that isotropic ceria nanoparticles are not capable of forming a rigid reinforcing network which would withstand a mechanical load, just like in nanocomposites filled with CNF [43,44] or CNT [45]. On the other hand, strong interactions between the polymer and the nanoparticles would probably result in an increase in the samples’ stiffness, the nanoparticles acting as links between the PI macromolecules. Such an effect has been reported for polymers filled with fullerenes [46]. Interactions between CeO_2_ nanoparticles and polymer matrices have also been registered [17,21,22]. However, in all these papers, a positive effect of the nanoparticles on the mechanical properties of nanocomposites was observed only at CeO_2_ concentrations below 1 wt% (less than 0.2 vol%), while at greater contents of the nanofiller the opposite effect was seen. As mentioned above (see Figure 2), the optimal CeO_2_ concentration providing the highest thermal stability for the DPhO-BAPS was 0.5 vol% (~3 wt%). Considering the stress-strain curves of both the PMDA-ODA and the DPhO-BAPS nanocomposites filled with the nanoceria only, along with their TGA curves (see Figure 5) and TMA data (Table 2; Table 3), one might conclude that there were no strong interactions between the polymer matrices and nanoceria to exist, at least at room temperature. Moreover, such a large amount of the nanofiller could cause the formation of aggregates, which, acting as stress concentrators, may worsen the mechanical properties of a material. 

As follows from Figure 8, as well as from Table 4 and Table 5, both types of carbon nanoparticles used in our experiments improved some of the mechanical characteristics of both PIs. For instance, the CNC increased the Young’s modulus by 15%, while the CNF augmented the same parameter by 11% for the PMDA-ODA. For the DPhO-BAPS, these characteristics became ~12% and 20% greater after doping the PI matrix with the CNC (3 vol%) and the CNF (5 vol%), respectively. In the case of CNC, such an effect can stem from the joint action of the following factors. On one hand, these nanoparticles were capable of forming a rigid framework within a polymer, (the CNC content in our experiments surpassed an estimated value of the percolation threshold [47]). On the other hand, the observed effects may well be explained by specific interactions between the CNC surface layer with aromatic rings and/or heterocycles of the PIs’ macromolecules. Similar results were obtained in [26], in particular, for PMDA-ODA. Moreover, the augmentative effect of CNC on nanocomposite stiffness has been confirmed by means of simulation-based modelling [48]. As for the CNF, as discussed above, the most probable mechanism for improving the mechanical properties of the PIs after filling them with these nanoparticles is the formation of a network structure, which reinforces a polymer. Taking into account the complex surface structure of CNF [49], the π-π stacking between their surfaces and PI macromolecules seems doubtful or, at least, less pronounced in comparison with CNC-PI interactions. Upon addition of 3 vol% of CNF in the DPhO-BAPS matrix, the Young’s modulus apparently stays intact. One could attribute this to a lack of nanoparticles involved in the formation of the rigid network [25].

Comparing the mechanical properties of the PIs containing only nanoceria and those doped with binary CeO_2_/nanocabon filler, one can notice that the Young’s moduli of the latter nanocomposites turned out to be much higher. Apparently, the negative effect of the CeO_2_ was compensated by the carbon nanoparticles. The best results were obtained for the DPhO-BAPS, the PI filled with the binary CeO_2_/CNC or CeO_2_/CNF mixtures having stiffness values comparable to those of the DPhO-BAPS/nanocarbon compositions, exceeding the stiffness of the pristine PI by almost 15%. It should be mentioned that, according to Table 1, such compositions, i.e., DPhO-BAPS/CNF/CeO_2_ and DPhO-BAPS/CNC/CeO_2_, had their thermal stability indices, *τ*_5_, enhanced by 16 °C and 19 °C with regard to the pristine polymer.

## 4. Conclusions

Two PI matrices, namely PMDA-ODA and DPhO-BAPS, were filled with binary nano-additives: either CeO_2_/CNF or CeO_2_/CNC. The thermal, mechanical, and thermomechanical properties of these nanocomposites were investigated and compared with those of the pristine PIs, as well as with the properties of these PIs doped with only one type of nanoparticles. We have found that the use of a binary filler could lead to simultaneous improvement of several groups of properties. For example, although the thermal stability of the PMDA-ODA/CNF/CeO_2_ composite was much lower than that of the pristine PI, it was established that the binary CeO_2_/CNF nano-additive provided a substantial increase in its stiffness above the glass transition temperature. Such a filler could be used where there is a need for the widening of a temperature range in which the PI would maintain its mechanical properties. As for the DPhO-BAPS, it has been shown that its nanocomposites containing binary CeO_2_/nanocarbon fillers were characterised not only by thermal stability values surpassing that of the pristine PI by almost 20 °C but also by stiffness values that were more than 10% higher than that of the matrix polymer. 

Thus, in this research, we developed a novel approach to conjoint optimization of various physical properties (particularly, thermal and mechanical) of thermally stable polymer materials, viz aromatic PIs. To this end, a preparative method was worked out, which had not been previously reported on, and included doping a PI matrix with a binary blend of nanofillers having different nature and acting synergistically with respect to the properties of interest. 

Summing up, the functional properties of PI-based nanocomposites containing a binary CeO_2_/nanocarbon filler can be tuned by varying their composition, structure and morphology. A choice of relevant dopants (binary or even ternary) and the optimal composition of polymer-based systems as a whole would ensure improved performance of the material, thereby extending its functionality.

## Figures and Tables

**Table 1:** Thermal stability indices of polyimides (PIs) and PI-based nanocomposites.

Sample	*τ*_5_, °C	*τ*_10_, °C
DPhO-BAPS	483	510
DPhO-BAPS/CeO_2_	511	539
DPhO-BAPS/CNF(3 vol%)	491	520
DPhO-BAPS/CNF(3 vol%)/CeO_2_	503	532
DPhO-BAPS/CNF(5 vol%)	478	504
DPhO-BAPS/CNF(5 vol%)/CeO_2_	499	528
DPhO-BAPS/CNC(3 vol%)	494	524
DPhO-BAPS/CNC(3 vol%)/CeO_2_	502	533

PMDA-ODA	519	542
PMDA-ODA/CeO_2_	451	472
PMDA-ODA/CNF(5 vol%)	524	546
PMDA-ODA/CNC(3 vol%)	529	553
PMDA-ODA/CNC(3 vol%)/CeO_2_	451	473
PMDA-ODA/CNF(5 vol%)/CeO_2_	454	477

**Table 2:** Thermomechanical characteristics of PMDA-ODA (PI with a repeating unit based on pyromellitic dianhydride (PMDA) and oxydianiline (ODA)) and its nanocomposites.

Sample	*T*_g_, °C	*ε*, %		
		*ε_T_*_g_^1^	*ε*_max_^2^	Δ*ε* = *ε*_max_ − *ε_T_*_g_
PMDA-ODA	397	2.9	15.6	12.7
PMDA-ODA/CeO_2_	397	2.6	12.2	9.6
PMDA-ODA/CNC(3 vol%)	398	2.5	7.7	5.2
PMDA-ODA/CNF(5 vol%)	394	2.4	6.0	3.6
PMDA-ODA/CNC(3 vol%)/CeO_2_	395	2.4	8.7	6.3
PMDA-ODA/CNF(5 vol%)/CeO_2_	394	2.7	5.8	3.1

^1^*ε_T_*_g_ were determined using intersection points of tangents of straight-line regions of the *ε*(T) functions. ^2^ *ε*_max_ were maximal deformations registered in the experiments.

**Table 3:** Thermomechanical characteristics of DPhO-BAPS (PI with a repeating unit based on 2,3,3′,4′-diphenyl ether tetracarboxylic acid dianhydride (dianhydride DPhO) and 4,4′-bis(4″-aminophenoxy)biphenyl sulfone (diamine BAPS)) and its nanocomposites.

Sample	*T*_g_, °C (DTA)	*T*_g_, °C (TMA)	*T*_fl_, °C ^1^
DPhO-BAPS	257	264	288
DPhO-BAPS/CeO_2_	255	262	284
DPhO-BAPS/CNC(3 vol%)	255	260	287
DPhO-BAPS/CNC(3 vol%)/CeO_2_	255	260	288
DPhO-BAPS/CNF(5 vol%)	255	258	294
DPhO-BAPS/CNF(5 vol%)/CeO_2_	254	260	293

^1^*T*_fl_ is the temperature at which a sample starts «flowing».

**Table 4:** Mechanical properties of the PMDA-ODA-based compositions.

Sample	*E*, GPa	*σ*_y_, MPa	*σ*_b_, MPa	*ε*_b_, %
PMDA-ODA	2.6	103	145	90
PMDA-ODA/CeO_2_	2.1	77	95	39
PMDA-ODA/CNC(3 vol%)	3.0	102	113	36
PMDA-ODA/CNC(3 vol%)/CeO_2_	2.3	80	95	38
PMDA-ODA/CNF(5 vol%)	2.9	90	114	44
PMDA-ODA/CNF(5 vol%)/CeO_2_	2.5	78	98	30

**Table 5:** Mechanical properties of the DPhO-BAPS-based compositions.

Sample	*E*, GPa	*σ*_y_, MPa	*σ*_b_, MPa	*ε*_b_, %
DPhO-BAPS	2.6	104	104	47
DPhO-BAPS/CeO_2_	2.4	-	90	12
DPhO-BAPS/CNC(3 vol%)	2.9	-	97	10
DPhO-BAPS/CNC(3 vol%)/CeO_2_	2.9	-	90	8.5
DPhO-BAPS/CNF(3 vol%)	2.6	80	76	12
DPhO-BAPS/CNF(3 vol%)/CeO_2_	2.8	-	89	11
DPhO-BAPS/CNF(5 vol%)	3.1	91	95	11
DPhO-BAPS/CNF(5 vol%)/CeO_2_	3.0	-	84	9.0
